# Addressing social responsibility of kidney healthcare facilities in Africa

**DOI:** 10.3389/frhs.2025.1523196

**Published:** 2025-05-14

**Authors:** Intissar Haddiya, Sara Ramdani, Wijdane Chtioui, Abdellatif Sidi Aly, Sidi Mohamed Mah, Abdou Niang, Awatef Azzabi, Ahmed Yahia Elmowafy, Maimouna Mahamat, Angélique Akagah Ademba, Sylvain Pierre Nzeyimana, Mahamat Abderraman G. Zalba

**Affiliations:** ^1^ Department of Nephrology, Faculty of Medicine and Pharmacy of Oujda, University Mohammed Premier, Oujda, Morocco; ^2^ Laboratory of Epidemiology, Clinical Research and Public Health, Faculty of Medicine and Pharmacy of Oujda, University Mohammed Premier, Oujda, Morocco; ^3^ Department of Nephrology-Hemodialysis-Polycinique Medipole, National Council of Donation and Transplantation, Nouakchott, Mauritania; ^4^ Department of Nephrology Hemodialysis and Transplantation, Centre Hospitalier National de Nouakchott, Faculty of Medicine, Pharmacy and Ondontostomatology, Nouakchott, Mauritania; ^5^ Department of Nephrology, Faculty of Medicine, Dakar University Medical Hospital, University Cheikh Anta Diop, Dakar, Senegal; ^6^ Department of Nephrology, Faculty of Medicine Ibn El Jazzar, Sahloul Hospital, University of Sousse, Sousse, Tunisia; ^7^ Nephrology Unit, Urology and Kidney Transplantation Center, Faculty of Mansoura, Mansoura University, Mansoura, Egypt; ^8^ Department of Nephrology, Faculty of Medicine and Biomedical Sciences, University of Yaoundé, Yaoundé, Cameroon; ^9^ Service de Néphrologie - Caisse Nationale de Sécurité Sociale, Université des Sciences de la Santé, Libreville, Gabon; ^10^ United Nations Clinic Kinshasa, United Nations Development Programme, Kinshasa, Democratic Republic of Congo; ^11^ Department of Nephrology, La Renaissance University Hospital, Faculty of Human Health Sciences, University of N'Djaména, N'Djamena, Chad

**Keywords:** Africa, kidney disease, public hospitals, social responsibility in health, nephrology

## Abstract

Social responsibility (SR) in health is of paramount importance in the development of health systems. It is, therefore, frequently presented as one of the solutions for healthcare improvement in most developed countries. In addition, SR in health has been the subject of several publications in the West and Asia. However, the absence of African data on this subject motivated us to carry out this study which aimed to assess SR of African public hospitals in terms of kidney disease management, and to explore the factors that influence it based on the perceptions of patients and nephrologists, and to suggest concrete solutions to improve it.

**Material and methods:**

Our study included participants, nephrologists and patients, of public hospitals in ten African countries. Two structured questionnaires were developed to measure the perception of patients and nephrologists regarding the hospital's performance based on four dimensions: Quality of service, pertinence of care, accessibility and professional ethics. Statistical analysis of the survey data included the Student's *t*-test, the Chi-square test, univariate and multivariate logistic regression methods on several levels, in order to examine the factors influencing the patients’ and nephrologists’ evaluation of hospital SR.

**Results:**

Only 26.95% of the included patients believed that their hospitals were socially responsible. In fact, the general conditions of the hospitals and the accessibility of care (waiting times, overall cost, unavailability of treatments) were generally perceived to be unsatisfactory. Also, patients generally believed that receiving treatment regardless of their ability to pay was not always possible.

As for doctors, 60% of the participants considered their hospitals not socially responsible. They had raised, in addition to the social determinants of health, a number of factors hindering SR of African hospitals, such as the lack of health professionals and brain drain.

Finally, nephrologists suggested solutions, mainly directed at hospital managers, encompassing the following three components: Recruitment and training of human resources, leadership and governance, as well as strengthening of infrastructures and logistics.

**Conclusion:**

Our study highlighted the crucial role of hospital governance and stakeholder involvement in improving SR and care delivery. Participants’ perceptions and concerns inform health authorities about managing kidney disease in African hospitals.

## Introduction

Social responsibility (SR) in healthcare has gained global recognition over the past decade, especially in developing nations and transitional societies. This emphasis underscores the critical role of health systems in enhancing access to and quality of care, particularly in underserved regions. In fact, it is now widely acknowledged that health status is influenced not only by genetic and physiological factors but also by a range of demographic, cultural, socio-economic, ecological, and health-related determinants that shape population health and life expectancy (1). Social responsibility is an ethical commitment by individuals or organizations to take actions that enhance the well-being of society (2). Healthcare SR encompasses policies and practices that aim at equitable resource distribution, community involvement, and improved healthcare accessibility (3, 4).

Past research on SR in healthcare indicates substantial improvements in patient satisfaction, trust, and health outcomes when SR principles are integrated. However, most studies in this area are from high-income countries, with limited data from Africa. The existing African studies did emphasise the need for community-centered healthcare models that leverage local resources and cultural understanding to increase healthcare accessibility and trust among underserved populations (5, 6). They also advocate that SR-focused policies should prioritize equitable resource allocation in low-income and rural areas (7, 8). Furthermore, they also discuss the need to enhance infrastructure and capacity-building by training healthcare professionals locally, in order to foster expertise and create sustainable, socially responsible healthcare systems (9).

Nonetheless, African studies on the topic remain scarce, highlighting the need for larger comprehensive studies regarding healthcare SR across Africa, so that African patients can benefit from medical progress and lower the disparities in healthcare access (10). A great example of these disparities is the inadequacies in healthcare management of kidney disease in our regions due to the shortage of healthcare structures, human and material resources needed for the management of kidney disease, as well as the lack of socially responsible healthcare systems which consider the social determinants of African populations as an integral part of patient management (11, 12). Therefore, we decided to design an African survey with the aim of assessing the patients’ and caregivers’ perceptions of SR of public hospitals. Specifically, our study seeks to explore the factors influencing healthcare SR from the perspectives of both patients and doctors and to suggest concrete solutions to address it.

### African context

Africa is a vast continent occupying 20% of the global land surface (13, 14). The age structure of Africa's population is young, with the fast growth of the age range 25–64. Moreover, according to data from the World Bank Group, the life expectancy in Sub-Saharan Africa was 60.4 years in 2015 and rose to 64.1 years by 2020 (15, 16).

However, the proportion of poor people in Africa continues to rise. It is worth noting that the health index among countries rises as their Gross Domestic Product (GDP) increases, which explains the inequalities between African countries in terms of the quality of healthcare and life expectancy (17).

The major causes of morbidity and mortality in Africa are communicable diseases such as HIV-AIDS and diarrheal diseases, as well as non-communicable diseases (NCDs) such as kidney and cardiovascular diseases, cancer and diabetes (17, 18).

The healthcare activity in Africa includes different entities, including faith-based and charitable institutions, public hospitals, private sector often located in high-income urban areas, and informal health sector including traditional healers and non-approved medicinal practices more famous among the low-income rural populations (19).

The overall score of essential services availability in Africa is extremely low, 0.36 on average, focused especially on communicable diseases’ control (0.76) (17). In addition, there are significant inequalities in access to these interventions not only between countries but also within each country.

### Nephrology in Africa

Nephrology has historically received limited attention within the African healthcare system due to competing health priorities. However, significant advancements have been made in the field over the past three decades, largely driven by the efforts of the African Association of Nephrology (AFRAN) and the International Society of Nephrology (ISN). These organisations have played a pivotal role in enhancing nephrology training and research across the continent (20).

Many reports have shown that the number of chronic kidney disease (CKD) patients and patients on dialysis is steadily increasing in Africa, however the incidence of acute kidney failure (AKF) is very variable. A study from Nigeria has found that AKF accounted for 35% of all admissions in Lagos over a 10-year period, other researchers found that it represented 4.5% of all admissions in Dakar and 23.2% of all Intensive Care Units (ICU) (21, 22). Records have shown that severe infection is the major cause of AKF in contrast to developed countries. HIV comes in first line. Other less reported but common causes in Africa include nephrotoxic plants, obstetric complications and various forms of envenomation (23, 24). Mortality due to AKF is very high in Africa, ranging from 13.3% to 79.8%. This is especially due to the lack of access to dialysis, which is self-funded without government assistance in many parts of the continent (22, 25).

The lowest prevalence of CKD is 4% (3%–13%) reported in North Africa, while the highest prevalence, 16.5%, (2%–41%) is observed in West and Central Africa, with the major cause being hypertensive vascular disease, followed by diabetic nephropathy (22, 26).

Regarding the treatment options for end-stage kidney disease (ESKD) in Africa, haemodialysis represents the most commonly utilised renal replacement therapy for ESKD, predominantly accessible in urban centres. However, the development and expansion of this modality are significantly hindered by widespread poverty and resource mismanagement in many countries across the region. Additional challenges include the limited availability of vascular surgeons to establish permanent vascular access, as well as inadequate infrastructure, such as restricted access to reliable water, electricity, and transportation services (27).

Peritoneal dialysis (PD) is theoretically the ideal option for patients in large rural areas. Unfortunately, the importation and transport of PD fluids increase the cost of this technique. In addition, overpopulation, lack of adequate water and sanitation and low levels of education, as well as the shortage of well-trained PD staff, make this therapy impractical. Even in the wealthiest countries of North Africa, only 3% of all dialysis patients benefit from this method (28).

Besides, Egypt, Sudan and South Africa are the leading African countries in terms of kidney transplantation. The major obstacles to the expanding of this treatment are the high cost, the lack of infrastructure and ethnic and religious beliefs regarding transplantation from deceased donors.

Therefore, kidney disease is an important public health problem, particularly in sub-Saharan Africa, many patients die without adequate care due to the shortage of nephrologists and health staff across the continent and a lack of resources.

## Material and methods

We conducted an African wide survey over a one-year period from February 2019 to February 2020, including African patients and nephrologists working in public regional and/or university hospitals with clinical nephrology, dialysis and/or renal transplantation services.

Based on suggestions from experts and findings from previous studies, we narrowed our research to the SR of hospitals from a ‘professional responsibility’ perspective.

The study population included nephrologists and patients diagnosed with kidney disease who were receiving care through different treatment modalities, including conservative management, dialysis (hemodialysis or peritoneal dialysis), and renal transplantation. Nephrologists included in the study were those actively providing nephrology care in public hospitals, ensuring that the responses reflected the experiences of healthcare professionals engaged in clinical nephrology, dialysis, and transplant services.

Key dimensions assessed in the study were defined as follows:

- Quality of care was defined as the ability of healthcare services to provide effective, safe, and patient-centered treatment in accordance with medical guidelines. Factors assessed under this dimension included timeliness of care, adherence to clinical protocols, and the quality of patient-provider communication.
- Pertinence of care referred to the appropriateness and necessity of the medical interventions provided, with specific evaluations focusing on diagnostic accuracy, the suitability of prescribed treatments, and alignment with patient needs.
- Accessibility was defined in terms of the ease with which patients could obtain healthcare services, including geographical proximity to healthcare facilities, financial affordability, and the availability of essential services such as nephrologists, dialysis, laboratory tests, and transplant-related care.
- Ethics, as another core dimension, was determined based on adherence to ethical principles such as patient confidentiality, informed consent, non-discrimination, and professional integrity in nephrology care.

Two structured questionnaires were developed to measure the perception of patients and nephrologists regarding the hospital's performance based on four dimensions: Quality of service, pertinence of care, accessibility and professional ethics. Each of these items was measured using a 5-point Likert scale. Moreover, the participating doctors were asked to answer open-ended questions aimed at better measuring their perception of hospitals’ SR in terms of kidney disease management, and also about the barriers to SR in their institutions and were invited to propose practical solutions. The reliability of the questionnaire was evaluated using Cronbach's alpha index, which ranged from 0.8 to 0.87 among patients and doctors. The internal consistency of the questionnaire was therefore good.

Statistical analysis of the survey data included the Student t-test, the Chi-square test, univariate and multivariate logistic methods at several levels, in order to examine the factors influencing the patients’ and nephrologists’ evaluation of hospital SR. A 95% confidence interval (CI) was defined for all estimates and a value of *p* < 0.05 was considered significant.

To address missing data and non-responses in the survey, a systematic approach was implemented to maintain data integrity and reduce bias. Data imputation using average values from respondents with similar demographics or regional backgrounds was employed, while variables with high non-response rates were excluded from specific analyses to ensure robustness. Data weighting adjustments were applied to account for disproportionate non-responses among certain demographics, and sensitivity analyses were conducted to assess the impact of imputation on results.

Our study was conducted in accordance with the ethical principles outlined in the Declaration of Helsinki, which sets forth guidelines to ensure respect for participants’ rights, integrity in the research process, and ethical responsibility in conducting human research. Ethical approval for the study was granted by the Ethics Committee of Medical Research at University Mohammed I, Oujda, Morocco, prior to data collection in 2019, and was subsequently amended in 2023 under approval number 13/2023. To ensure ethical rigour, the research adhered to a comprehensive set of moral principles that protected the rights and welfare of all participants. Participants were clearly informed of their rights, including the voluntary nature of their involvement, and were made aware that they could withdraw from the study at any time without any negative consequences.

The informed consent form provided detailed information about the study's objectives, procedures, and the confidentiality of personal data. It emphasised that all data would be anonymised and used solely for research purposes. Efforts were made to ensure that participants comprehended all aspects of the study, allowing them to make informed decisions regarding their participation. This approach reflects the study's commitment to upholding ethical standards in research and ensuring participants’ autonomy, confidentiality, and protection throughout the study.

## Results

### Patients: characteristics and perceptions of hospital SR

#### Patient demographics and socioeconomic characteristics

Our study included 310 patients from the five African regions, North Africa (43.87%), Central Africa (25.81%), West Africa (16.13%), Southern Africa (5.48%) and East Africa (8.71%). The targeted countries can be classified by income into three categories, upper-middle-income countries (Gabon), lower-middle income countries (Morocco, Tunisia, Egypt, Senegal, Mauritania, Cameroon) and low-income countries (Chad, Burundi, Mozambique) (29).

The mean age in our study was 46.51 ± 14.67 with extremes ranging from 18 to 84 years of age. 90.73% of the participants were under 65. Our patients were predominantly male with a male-to-female sex ratio of 1.42, except for Southern Africa, Cameroon, Mauritania, Mozambique and Tunisia where a female predominance was noted. In total, 74.7% of the included patients in our study lived in urban areas.

#### Economic and educational profiles

46.26% of the surveyed patients reported having a low income, 43.54% had a medium income, and only 10.20% had a high income. In our study, 64.77% of patients benefited from health coverage, mainly in North Africa; however, 64.7% of the participants from Southern Africa, 55.1% in Central Africa, and 69.6% in West Africa, had no health coverage. Furthermore, 24.23% of the patients included in our study had been to university (mostly from Southern Africa), 31.40% attended secondary school (mostly from Central and North Africa), 21.16% attended primary school (mostly from East Africa), and 23.21% were illiterate (mostly from West Africa).

#### Healthcare and satisfaction

Our study was conducted in public hospitals, most of which were of medium size (75.08%). One of the main criteria was the existence of at least one nephrologist and a specialised care unit for kidney disease. The majority of our patients had chronic renal failure 83.6%, compared with 16.4% with acute kidney failure. A total of 56.1% of these patients had only eight hours of dialysis a week instead of the 12 h a week usually recommended. None of the patients participating in our study underwent PD, and only 2.59% had a kidney transplant, mainly Egyptians (36.8%) and Tunisians (6.3%). Regarding haemodialysis vascular access, 75.42% of the chronic haemodialysis patients had a permanent vascular access, arteriovenous fistula (AVF) while the remaining 24.58% had a temporary venous catheter (mostly in West Africa).

Our patients were generally satisfied with the clinical examination (56.7%), the doctor's prescriptions (59%), and treatment effects (58.4%). As for satisfaction with the care process, including staff attention, updates about the disease, treatments and their side effects, patients were less satisfied (only 49.1% expressed satisfaction), and 46.8% remained neutral. The highest satisfaction rate was reported in Southern Africa (47.1%).

Regarding people's perception of the quality of communication, information and coordination of caregivers, the patients included in our study were mostly neutral. As for their satisfaction with the environment and general conditions of the hospital setting, 45.8% were neutral, 19.7% were very satisfied (Cameroon and Tunisia for instance) and 10.6% were dissatisfied.

In terms of access to health care, 46.6% of patients were neutral about waiting times for consultation or hospitalisation, 19.4% were satisfied, 17.2% very satisfied, mostly in South Africa, Cameroon, Tunisia and Mozambique and 10% dissatisfied. As for the availability of free-of-cost medicines, 41.7% of patients were neutral, 21.8% were satisfied, 11.7% very satisfied, 15.3% dissatisfied and 9.4% very dissatisfied predominantly in West Africa. Likewise, concerning patients’ satisfaction with the overall healthcare costs (treatment, laboratory tests, and medical imaging), 45.1% of patients were neutral and 20.2% were dissatisfied. In Morocco for example, dissatisfaction was rated at 32.7%.

Medical practice requires a high level of ethical standards. In our study, the ethical dimension was investigated through the following items: equity in access to health care, opportunity for treatment regardless of a patient's ability to pay, and respect for patient privacy. Regarding the perception of equity in access to health care, 49% of patients were neutral, 22.1% and 23.4% were satisfied to very satisfied respectively, and 5.5% were dissatisfied. Satisfaction was noted especially in North and West Africa. Furthermore, the majority of the participants reported that receiving care regardless of their ability to pay is possible, while only 3.6% of the patients denied this. Finally, concerning the patients’ satisfaction with the measures taken to protect their privacy, 47.9% were neutral, 30.6% and 18.6% were satisfied to very satisfied, and just 3% were dissatisfied.

#### Perceptions of hospital SR

Regarding patients’ perception of hospital SR, 49.35% of them gave an average rating, 26.95% perceived it as good (Southern Africa, Senegal, Gabon), while 12.66% perceived it as poor. The patients’ perception of hospital SR was negatively associated with accessibility of healthcare (cost of treatment *p* = −0.003; 95% CI 0.16–0.25) and positively related to professional ethics (privacy *p* = 0.039; 95% CI 0.01–0.04). In addition, our analysis proved that there is a statistically significant correlation between the patients’ perception of hospital SR and some sociodemographic data, such as the African region (patients from Southern and Central Africa have a better perception), the hospital size (with better satisfaction from patients treated in larger hospitals), residence (urban patients were less satisfied than rural ones), patient type (inpatients were more satisfied than outpatients), and, finally, the availability of health coverage. However, there was no statistically significant connection between people's perception of SR and age, gender, personal income, or education level (Table 1). On the other hand, our study demonstrated that there is a significant positive association between the patients’ perception of hospital SR and the country's income (*p* < 0.001, 95% CI 0.06–0.12), as well as the country's health expenditure (*p* = 0.011; 95% CI 0.11–0.19).

**Table 1:** Patients’ characteristics and perception of hospital social responsibility.

Characteristics	Overall (*n* = 310)
5 African regions:	
- North Africa	43.87%
- Central Africa	25.81%
- West Africa	16.13%
- Southern Africa	5.48%
- East Africa	8.71%
Mean age ± SD	46.51 ± 14.67
M/F sex ratio	1.42
Patients’ income	
- Low income	46.26%
- Medium income	43.54%
- High income	10.20%
Health coverage	
- Yes	64.77%
- No	35.23%
Education	
- University	24.23%
- Secondary school	31.40%
- Primary school	21.16%
- Illiterate	23.21%
Kidney impairment	
- Chronic renal failure	83.6%
- Acute kidney failure	16.4%
- Kidney transplantation	2.59%
Hemodialysis vascular access	
- Permanent vascular access (AVF)	75.42%
- Temporary venous catheter	24.58%
People's satisfaction about	
Satisfied	
- Clinical examination	56.7%
- Doctor's prescriptions	59%
- Treatment effects	58.4%
Less satisfied with the care process	49.1%
Neutral	46.8%
Environment and general conditions of the hospital setting	
- Very satisfied	19.7%
- Neutral	45.8%
- Dissatisfied	10.6%
Access to health care	
- Very satisfied	17.2%
- Satisfied	19.4%
- Neutral	46.6%
- Dissatisfied	10%
- Very dissatisfied	68%
Availability of free-of-cost medicines	
- Very satisfied	11.7%
- Satisfied	21.8%
- Neutral	41.7%
- Dissatisfied	15.3%
- Very dissatisfied	9.4%
Perception of equity in access to health care	
- Very satisfied	23.4%
- Satisfied	22.1%
- Neutral	49%
- Dissatisfied	5.5%
Patients’ satisfaction with the measures taken to protect their privacy	
- Very satisfied	18.6%
- Satisfied	30.6%
- Neutral	47.9%
- Dissatisfied	3%
Patients’ perception of hospital social responsibility	
- Good	26.95%
- Average	49.35%
- Poor	12.66%

### Nephrologists: characteristics and perceptions of hospital SR

#### Demographics and professional attributes

Our study included 45 volunteer nephrologists from ten African countries, 55.56% from North Africa, 15.56% from West Africa, 4.44% from East Africa, 17.78% from Central Africa and 6.67% from Southern Africa. The mean age of the participating nephrologists was 38.84 ± 7.65 years, with extremes ranging from 26 to 58 years. The 30–40 age range was the most represented category. The mean professional seniority was 9.30 ± 5.83 years. The male-to-female sex ratio was 1.15 with 46.51% of female nephrologists. A total of 61.9% of the participating doctors were practising in regional public hospitals and 38.1% in the university sector, mainly in Morocco, Egypt, Tunisia, and Chad.

#### Perceptions of hospital environment and care process

**Figure 1:**
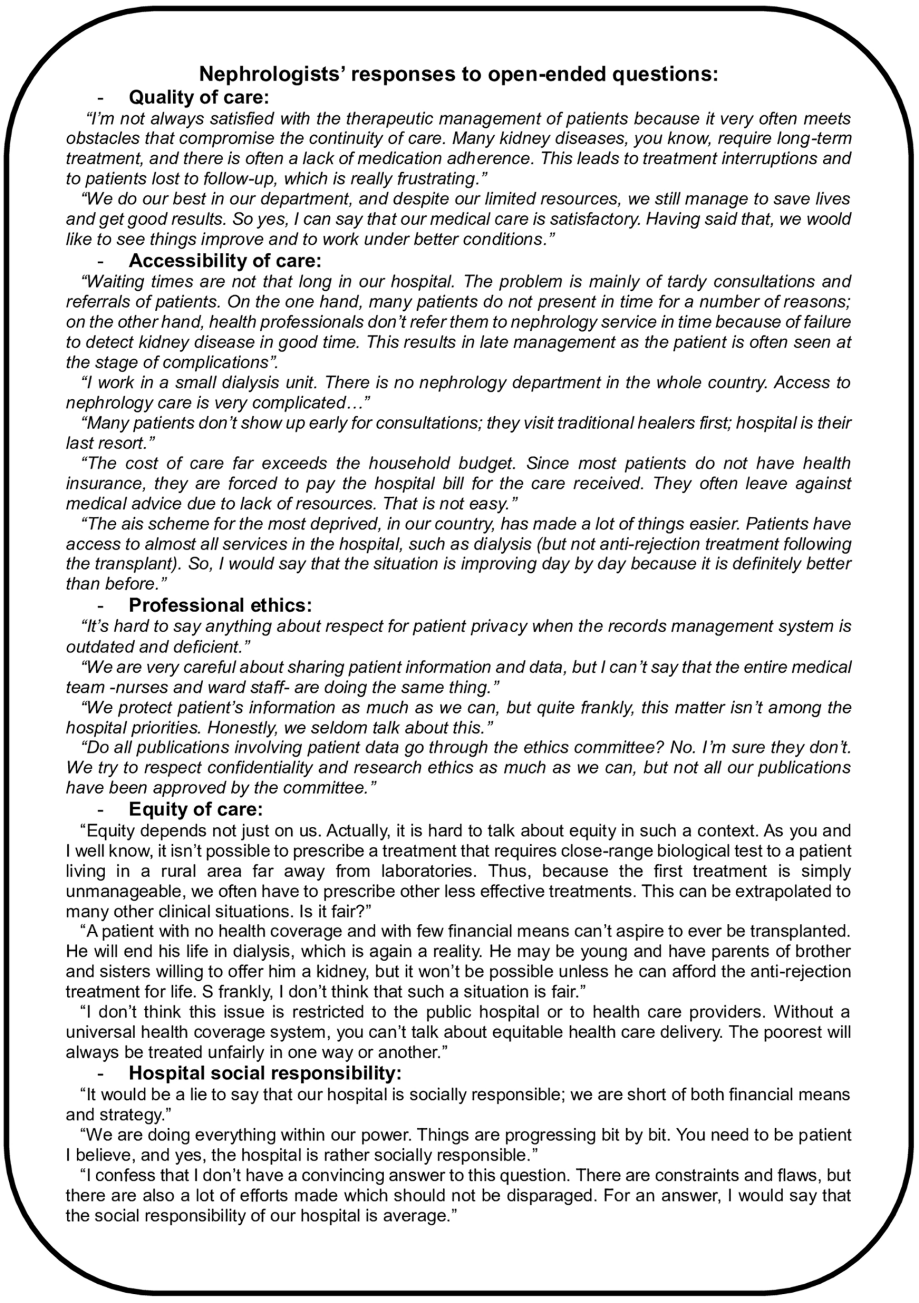
Voices of nephrologists: critical reflections on systemic challenges in kidney care.

Analysing doctors’ perception of hospital SR is crucial in order to improve the quality of care. Thus, we assessed doctors’ satisfaction with the constituent dimensions of this concept. The first dimension explored was their perception of the quality of care including therapeutic management, the environment and general conditions of the hospital settings and the care process. A total of 53.3% of the doctors were dissatisfied with the hospitals’ environment and conditions, 15.6% were very dissatisfied, 17.8% were neutral and 13.3% were satisfied. As for the doctors’ satisfaction with the care process, our study showed that 35.6% of them were dissatisfied (Burundi, Mauritania), 11.1% were very dissatisfied, 15.6% were neutral and 37.8% were satisfied (Egypt, Senegal and Tunisia). Accessibility of care was the second assessed dimension. Regarding waiting times, 33.3% of doctors expressed their satisfaction with the referral time of their patients to receive consultation or hospitalisation in their health facilities, 24.4% were dissatisfied, 26.7% were very dissatisfied, the rest were neutral. For instance, most nephrologists from Egypt were satisfied, in contrast; doctors from Burundi and Mozambique were mostly dissatisfied. Meanwhile, 24.4% of the doctors were satisfied with the cost of care applied by their hospitals, 37.8% were neutral, 20% were dissatisfied and 13.3% were very dissatisfied. Concerning the availability of treatment in hospitals, dissatisfaction was predominant, with 35.7% of doctors were dissatisfied and 23.8% were very dissatisfied except for Egypt and Morocco. All due to the low investment and lack of strategic health financing policies (Figure 1).

#### Medical confidentiality

Moreover, the appreciation of participating doctors about hospitals’ professional ethics was also evaluated. Medical confidentiality was assessed first, 40% of doctors were satisfied with the measures taken by their hospitals to protect patients’ privacy, 35.6% were neutral and 24.4% were dissatisfied. Many African nephrologists stated through open-ended questions the poor quality of record management, the lack of awareness among all stakeholders about the value of the confidentiality and manifested their concern about the delicate situation of scientific publishing without prior consent of patients. About the doctors’ perceptions of equity in healthcare delivery, responses were variable. A total of 44.4% of nephrologists felt that equity was only “sometimes” respected in the management of their patients, 17.8% reported that their patients were treated fairly and 6.7% said that it was rarely the case. In fact, most of the interviewed doctors stated that they were doing their best to provide care regardless of their patients’ socio-economic level. In fact, only 15.6% of doctors included in our study confirmed the ability of their hospitals to provide healthcare regardless of the patients’ capability to pay (Egypt).

Finally, regarding hospital SR, 60% of practitioners thought that their hospitals were not socially responsible, 28.89% thought that their hospitals’ SR was average such as Tunisian and Egyptian doctors, and 8.89% stated that it was good, mainly from Senegal.

In summary, the average scores revealed that African doctors were generally dissatisfied with both dimensions examined (quality and accessibility of care, and professional ethics) as well as with their hospitals’ SR. Our study revealed that there is a significant positive correlation between doctors’ perception of hospital SR and accessibility of healthcare (cost of treatment *p* = 0.039; 95% CI 0.03–0.23), no other significant correlation was proven. Furthermore, statistical analysis of our study showed no significant correlations between doctors’ perceptions of the hospital's SR and their socio-professional attributes nor with the country's income level (*p* = 0.262) or country health expenditure (*p* = 0.118) (Table 2).

**Table 2:** Nephrologists’ characteristics and perception of hospital social responsibility.

Characteristics	Overall (*n* = 45)
5 African regions	
- North Africa	55.56%
- West Africa	15.56%
- East Africa	4.44%
- Central Africa	17.78%
- Southern Africa	6.67%
Mean age ± SD	38.84 ± 7.65 years
Sex ratio M/F	1.15
Participating doctors	
- Regional public hospitals	46.51%
- University sector	38.1%
Practice	
- Both clinical nephrology and hemodialysis	38.64%
- Only dialysis	20.45%
- Clinical nephrology	22.73%
Perception of the quality of care	
Hospitals’ environment and conditions	
- Satisfied	13.3%
- Neutral	17.8%
- Dissatisfied	53.3%
- Very dissatisfied	15.6%
Care process	
- Satisfied	37.8%
- Neutral	15.6%
- Dissatisfied	35.6%
- Very dissatisfied	11.1%
Waiting time	
- Satisfied	33.3%
- Neutral	15.6%
- Dissatisfied	24.4%
- Very dissatisfied	26.7%
Cost of care	
- Satisfied	24.4%
- Neutral	37.8%
- Dissatisfied	20%
- Very dissatisfied	13.3%
Medical confidentiality	
- Satisfied	40%
- Neutral	35.6%
- Dissatisfied	24.4%
Equity in health care delivery	
- Sometimes	44.4%
- Fairly	17.8%
- Rarely	6.7%
Hospital social responsibility	
- Good	8.89%
- Average	28.89%
- Not socially responsible	60%

In fact, the doctors’ perceptions of the SR of their hospitals were less positive than patients’ perceptions. The mean SR score reported by doctors was 2.46 (95% CI: 2.20–2.72), while that reported by patients was significantly higher, at 3.49 (95% CI: 3.41–3.57), with this difference being statistically significant (*p* = 0.05). This could be explained by the fact that patients were probably cautious or even distrustful.

## Discussion

### Patients

Despite the support of many nephrologists, our study only managed to include 310 patients. The three most commonly reported barriers are (30, 31):

- Lack of motivated workforce due to the overwhelming workloads. In fact, sub-Saharan Africa has less than 3% of the world’ health workforce.
- Financial barriers and logistical challenges for collecting/submitting data.
- Regulations at the national or hospital level including the approval of ethics committee.

Most of the patients included in our study were young with a mean age of 46.51 ± 14.67. These results differ significantly from the literature data and particularly Western sources which confirmed the high prevalence of CKD among the elderly (32). According to an American survey (NHANES), more than a third of people over 70 years have moderate or severe CKD (33). Gender is a well-known determinant of the risk of development and progression of CKD, which tends to be higher in women. In Europe, for example, the male-to-female sex ratio is 0.96 (34). However, our study showed a predominance of men participants with a male-to-female sex ratio of 1.42. This goes hand in hand with a French study on dialysis patients’ satisfaction showing that 60.3% of participants were men, which was explained by the fact that women tend to be less responsive to questionnaires (35). Many African studies stated that African patients were more likely to seek healthcare at public health facilities. Thus, our study was conducted exclusively in public hospitals (36). 61.5% of our participants were chronic haemodialysis patients, thanks to the special relationship built between the nursing staff/doctors and their patients at haemodialysis centres (37).

Regarding the patients’ satisfaction with the quality and pertinence of care, more than 50% of our patients were satisfied, our results are consistent with western studies (38). Nevertheless, the authors of a large American multicentric study recommended caution in interpreting these results, because only clinically stable patients take part in these surveys and dissatisfied patients often decline invitations to participate, which may represent a non-negligible selection bias (39). Furthermore, Weaver et al. noted that some other patients’ factors (age, educational level expectations and personal preferences) may influence satisfaction (40).

Dissatisfaction is often reported among patients with chronic diseases especially CKD and dialysis patients. A large multicentre study in Europe and South America examining the satisfaction of haemodialysis patients reported that in spite of patients’ satisfaction with the staff attention, they were deceived with the quality of information provided by the medical team regarding treatment modality, prognosis and the likelihood of a kidney transplant (41). Other western studies backed these same constatations, stating that misinformation and inadequate communication were the biggest concern of their patients (39). Indeed, half of adults with chronic diseases in Norway and Sweden complained about the lack of communication with their care givers concerning their care goals and priorities, or their treatment options (42). In contrast, most patients in our survey didn’t particularly raise these questions about communication and information. On the other hand, 45.8% of patients surveyed were neutral about their satisfaction with the environment and general conditions, 10.6% were dissatisfied and 5.8% were very dissatisfied. In fact, a WHO study conducted in Africa noted that poor infrastructure and the inadequate environment of healthcare facilities were among the factors behind patient dissatisfaction with health services (30). The lack of access to healthcare including waiting times, availability of medicines, and healthcare costs, is also one of the main factors of people's dissatisfaction, not to mention long travel distances, unaffordable transport systems, low quality of care, lack of qualified professionals and poor organisation (36, 43, 44). In our study about 45% of patients were neutral about access to health care, less than 20% were satisfied and the rest were dissatisfied. However, it should be noted that access to health is a widespread problem around the world. In fact, about half of Canadian, German and Norwegian adults stated that they could not get an appointment on the same day or on the following day. Moreover, 30% of Canadian adults reported waiting two months or more before seeing a specialist followed by Norwegian 28% (42).

As for professional ethics, neutrality was again noted, as that 49% and 47.9% of patients were neutral about their perceptions of equity in access to healthcare and privacy protection respectively, only a minority was dissatisfied, as well as for their ability to receive treatment regardless of their capacity to pay. These results concord with those of the WHO multicentric study, in which equity in healthcare is closely related to the implementation of universal health coverage permitting a full range of quality health services without the risk of financial hardships (36).

Regarding privacy, only few studies in Africa sought to determine the extent of privacy and personal data protection, but recently, due to the rapidly evolving global digital environment, privacy protection became an important issue, and so many laws have taken place (45).

Our study took a major interest in patients’ perception of hospital SR. This perception was average in 49.35%, good in 26.95%, poor in 12.66%. Our results were far from those of a Chinese study by Liu W et al, published in 2016, reporting that the majority of patients were very satisfied with the SR of the surveyed publics hospitals (46). Besides, Wu and Naidu showed that the quality of care has a positive impact on patient's satisfaction, loyalty and perception of hospital SR (47, 48). Moreover, an Indonesian study noted that patients’ perception was related to three main aspects: quality, pertinence and financial aspects (49). Although, this is quite different from our study's findings. In fact, our study didn't find a significant correlation with the quality of care but with the accessibility of care instead. Contrary to our results, a French survey found that the satisfaction level of dialysis patients was mainly dependent on age. However, there was no correlation to gender, which concords with our study (35). Furthermore, a Saudi study showed that higher levels of education were significantly correlated with better satisfaction rates (50). In addition, a large survey published in 2016, found that financial barriers hindered access to healthcare in developed countries, thus influencing their perceptions of SR. This was mainly due to differences in health insurance design (42). Unexpectedly these two correlations were not found in our study. In fact, the existence of health coverage was significantly correlated to patients’ satisfaction. Same as our study, an earlier African study conducted in Ghana noted that patients treated in larger health facilities were healthier and more satisfied due to the increased availability of medicine supplies (51). Finally, our study found a significant correlation between the country's income and health expenditure which is consistent with many other rigorous studies. In fact, a patient from a high-income country is 3,400 times more likely to be satisfied with their country's healthcare system (52).

### Nephrologists

The mean age of nephrologists included in our study was 38.84 ± 7.65 years, with a mean professional seniority of 9.30 ± 5.83. This was observed in multiple publications. An Iranian study evaluating the perception of SR in hospitals found the mean age of participants who accepted to respond to the survey was 34.5 years. On the other hand, an Indian study examining the perception of doctors about hospital SR found that the third of respondents had a working experience less than 5 years (53, 54). The male-to-female sex ratio was 1.15 with 46.51% of female nephrologists. According to American Society of Nephrology, women constitute a reasonable proportion of the total workforce in nephrology (55).

A study sponsored by the American Medical Association reported that being able to provide high-quality healthcare is the primary motivation of doctor's professional satisfaction (56). The evaluation of the quality of care depends not only on therapeutic management, but also on the environment and general conditions of the hospital setting, and care process. In our study, most of the doctors were dissatisfied with their working environment. In fact, in many parts of the world, health professionals are exposed to a number of challenges such as inadequate infrastructure, overcrowded health services and administrative problems, especially in Africa where the lack of skilled healthcare workers and modern equipment constitute one of the main flaws in healthcare systems and a major cause of doctors’ dissatisfaction (57, 58). A satisfactory care process is the act of providing care in conformity with current scientific knowledge without any delays. Only 37.8% of the participating doctors were satisfied with the care process, the rest were either dissatisfied or neutral. This is due to the lack of medicines, imaging and biological tests required for diagnosis, treatment and follow up. Access to health is a major problem in developing countries especially for low-income patients (59). Starting with referral times, only one-third of the included doctors were satisfied. Other studies conducted in many developed countries (Canada, USA, UK, Australia) also reported longer waiting times especially in emergency departments, which leads to delays in the management of serious conditions and potentially adverse consequences (60). Late referral time is also problematic, in Douala, Cameroon. For instance, 3–4 CKD patients present late for nephrology care. As a consequence, 96% of late-referred patients required emergency dialysis on a temporary catheter which exposes them to multiple complications related to the use of this vascular access (61). As for the cost of care, only 24.4% of doctors in our study were satisfied. The low satisfaction rates of nephrologists with the cost of care in Africa are understandable because such care is not affordable and impedes the quality of care. Despite investments in public health, many developing countries are still in struggle. In Ecuador for example, out-of-pocket expenditure on health accounts on average 7.2% of total family income. Better-off families spend more on private consultations and insurance which counts for 71% of health expenditure, while the poorer ones pay more in out-of-pocket expenses for medicines which explains the widespread self-medication in these communities (57). Not to mention the non-availability of essential treatments in hospitals. In our study, 59.5% of nephrologists were dissatisfied with this matter. In fact, 50% of the population in the African region lacks access to essential medicines, according to a study by Kirigia JM et al., this is due to weak and under-resourced healthcare systems, because of the low investment, lack of strategic health financing policies, social security and weak coordination mechanisms (62).

Regarding professional ethics, privacy comes first, only 40% of doctors in our study were satisfied with the measures taken to protect patients’ privacy. Many African nephrologists reported the poor quality of record management in their hospitals, low interest in the value of confidentiality and lack of awareness. In the context of medical publications, respecting patients’ confidentiality while communicating the pertinent details of their cases is sometimes delicate (63). Medical journals have taken up this challenge by asking authors to obtain informed consent from patients. This procedure is imperative to help avoid the potential consequences of disclosing patient privacy (64). Equity in health in our context implies providing specialised nephrology care including costly replacement technologies in a resource-limited area. This aligns with the observations of an international group of experts who studied equity of access to healthcare for ESKD in Africa noting that equitable implementation of health programmes such as kidney replacement therapy requires considerations of three elements: availability, affordability and acceptability (65). Yet, low- and middle-income countries often lack financial, logistical and labor force capacity to provide such care, with a low participation of the public sector (66). Furthermore, it has been estimated that 188 million people across low- and middle-income countries experience catastrophic health expenditure annually due to kidney disease (58). Hence, in our study only 17.8% of doctors had a positive perception of equity in healthcare of their hospitals. Moreover, in terms of doctors’ perception of care delivery regardless of patients’ ability to pay, responses were particularly edifying. Despite the efforts made to provide care regardless of patients’ socio-economic level, the inequity gap between health service recipients and their income level continues to widen. Thus, low-income patients remain in waiting lists for dialysis due to their inability to afford access to private facilities. In addition, patients without health coverage cannot be transplanted because of their inability to pay for anti-rejection treatment (67).

Our study concluded that 60% of the included doctors were dissatisfied with their hospitals’ SR, 28.89% described it as average. Dissatisfaction is found in several publications about the perceptions of African doctors (68, 69). Meanwhile, an Iranian study identified a moderate level of satisfaction with a considerable difference in perceptions between managers and staff (53).

While examining the correlation between the dimensions of hospital SR and doctor's perceptions, we noticed that only one item relative to accessibility of care (cost of treatment *p* = 0.039) was positively correlated. In addition, our study showed no correlation between doctors’ socio-professional attributes and their perceptions of the hospital's SR. In contrast, an Indian study found that younger doctors tend to be more satisfied which decreases with longer time in service beyond five years, probably because the dynamics of the medical work environment usually dampen the initial enthusiasm (54). No correlation between countries’ socio-economic data and doctor's perceptions of hospital's SR was found.

Finally, our study revealed a statistically significant difference (*p* = 0.05) between doctors’ and patients’ perceptions with better scores among patients. Overall, patients were neutral in responding to several questions about the general conditions of the hospitals and care process while doctors were more commonly dissatisfied. As reported by some authors, usually patients, taking part in this kind of care satisfaction questionnaires, are the clinically stable and satisfied patients which may be a selection bias. Also, many patients worry about the potential negative consequences of their responses, particularly in the context of limited access to healthcare and chronic conditions such as dialysis which require frequent and prolonged contact with the same care facility and caregivers (38, 40).

Compared to Latin-American countries like Brazil—where universal healthcare provides free and widespread access to renal replacement therapies—the African context reveals more pronounced disparities in infrastructure, treatment availability, and legal protections for CKD patients (70). This contrast underscores the need for policy frameworks in Africa that not only address nephrology-specific gaps but also align with global standards in chronic disease management and health equity.

Current research specific to SR within African healthcare contexts reflects an urgent need to address these disparities. Reports by the WHO and regional health studies emphasise that SR is critical for achieving equitable health outcomes, yet African nephrology significantly lags behind global standards in SR-focused policies and investments (71). Factors such as inadequate funding, a shortage of specialised healthcare personnel, and limited policy frameworks contribute to these gaps. These challenges highlight the necessity for targeted policies and context-specific SR frameworks that consider Africa's socio-economic and healthcare infrastructure limitations.

The gaps identified in this study present opportunities for further research into SR-specific interventions tailored to the needs of African nephrology, such as developing sustainable SR policies, increasing nephrology training programmes, and implementing subsidised treatment initiatives. These efforts could bridge disparities in kidney healthcare across the continent, addressing both immediate and systemic barriers to equitable care. Addressing these gaps is essential not only for improving nephrology services in Africa but also for expanding scientific knowledge on effective SR models in low-resource settings.

## Conclusion/recommendations

While not representative of the entire population of kidney patients and nephrologists in Africa, our study had managed to assess perceptions and concerns of both patients and nephrologists in the aim to provide information to health authorities in order to improve healthcare policies and the quality of health care. Nevertheless, there are a number of limitations to our study. Our participants represent neither all stakeholders of healthcare system nor all African countries. In addition, our survey could not investigate the impact of patients’ clinical data nor was it able to assess possible links between these data and their perceptions of hospital SR. Moreover, it was easier to include chronic haemodialysis patients than other categories; as 61.5% of the patients who consented to participate in this study were chronic haemodialysis patients, as missing data from underrepresented patient groups may have introduced bias and limited the generalizability of the findings. Thus, the results should be interpreted with caution.

Further research in this field is needed before the results of the study can be generalised.

Besides, addressing SR in African nephrology care requires targeted strategies to improve quality, accessibility, and ethical practices. Recommendations based on the study's findings include increasing investment in hospital infrastructure, particularly for dialysis and nephrology units in rural and low-income areas, to ensure the availability of essential medicines, diagnostic tools, and access to haemodialysis and PD. Training programmes should focus on developing local expertise in nephrology, alongside incentives to retain healthcare professionals and mitigate brain drain from the continent.

Governance models grounded in SR principles are essential, promoting regular stakeholder engagement, transparent communication, and patient-centred policies aligned with ethical standards. Financial accessibility must also be addressed through subsidized or government-funded dialysis programmes, particularly in areas where patients currently bear the full cost of treatment. This would improve access for low-income patients and reduce mortality linked to delayed care. Hospitals must uphold high ethical standards to protect patient privacy and ensure equitable treatment, focusing on reducing socioeconomic disparities to foster trust and satisfaction.

## Data Availability

The raw data supporting the conclusions of this article will be made available by the authors, without undue reservation.
